# A Study on the Through-Plane Permeability of Anisotropic Fibrous Porous Material by Fractal Stochastic Method

**DOI:** 10.3390/ma15228064

**Published:** 2022-11-15

**Authors:** Yao Xu, Lianlian Xu, Shuxia Qiu, Zhouting Jiang, Binqi Rao, Peng Xu

**Affiliations:** 1 College of Science, China Jiliang University, Hangzhou 310018, China; 2 College of Mechanical and Electrical Engineering, China Jiliang University, Hangzhou 310018, China

**Keywords:** fibrous porous material, through-plane permeability, fractal, stochastic reconstruction, finite element method

## Abstract

The through-plane permeability is of great importance for understanding the transport phenomenon in anisotropic fibrous porous material. In this paper, a novel pore-scale model based on the equilateral triangle representative unit cell (RUC) and capillary bundle model is developed for the fluid flow through the anisotropic fibrous porous material according to fractal theory, and the effective through-plane permeability is presented accordingly. The digital structures of the fibrous porous material are generated by a fractal stochastic method (FSM), and the single-phase fluid flow through the 3D-reconstructed model is simulated by using the finite element method (FEM). It was found that the effective through-plane permeability depends on the fiber column size, porosity, and fractal dimensions for pore and tortuosity. The results show that the predicted through-plane permeability by the present fractal model indicates good agreement with numerical results and available experimental data as well as empirical formulas. The dimensionless through-plane permeability is positively correlated with the porosity and negatively correlated with the fractal dimensions for pore and tortuosity at certain porosity.

## 1. Introduction

Fibrous porous materials with advantages such as high specific area, high thermal conductivity, and good flexibility, etc., have been widely applied in textile fabric, fuel cells, filtration, and acoustic engineering, etc. [1,2]. Meanwhile, the conducting properties of fibrous porous material, including thermal conductivity and permeability as well as diffusion coefficient, strongly depend on its complex microstructures. Therefore, the characterization and reconstruction of microstructure and transport properties of fibrous porous material have attracted broad interests from multi-discipline fields [3,4,5].

Recently, with the development of computational technology, a few numerical reconstruction methods, including simulated annealing method (SAM) [6], Markov chain Monte Carlo (MCMC) [7], Bayesian reconstruction method (BRM) [8], random generation-grow method (RGGM) [9], multiple-point statistics method (MPSM) [10], etc., have been proposed to generate the digital structure of fibrous porous material, and a variety of simulation methods such as pore network modeling (PNM) [11], lattice Boltzmann method (LBM) [12], direct simulation Monte Carlo method (DSMC) [13], finite element method (FEM) [14], etc., have been applied to study the transport properties and mechanisms of fibrous porous material. Xie et al. [15] proposed a random-number-based algorithm to reconstruct the structure of fibrous silica aerogel and predicted its thermal conductivity by using discrete ordinate method and finite volume method (FVM). Hosseinalipour and Namazi [16] proposed a reconstruction algorithm based on the fiber orientation and explored the effect of geometric parameters of fibrous porous material on its transport property by FEM. Xu et al. [17] combined SAM with a hybrid function of two-point correlation function and lineal-path function as well as co-occurrence correlation function to reconstruct the 3D microstructures of two-phase fiber-pore hand sheets and simulated the absolute permeability based on computational fluid dynamics (CFD). Shi et al. [18] used stochastic orientation method to generate the fibrous structure of gas diffusion layer (GDL) and simulated the transport behavior of gas–liquid two-phase through the GDL by the volume of fluid (VOF) method. In order to explain why the transverse permeability of the fibrous porous material increases with the increase of through-plane angle of fibers, Pradhan et al. [19] generated a series of 3D fibrous structure by using the voxel model and calculated the transverse permeability by solving Stokes flow equations. Gostick [20] presented a novel algorithm to reconstruct pore networks from 3D fibrous porous material by using standard image analysis techniques and obtained the anisotropic permeability with a PNM tool. Wang et al. [21] developed a quartet structure generation set (QSGS) method to reconstruct the micro morphology of fibrous porous material and calculated the effective thermal conductivity based on LBM. Zhang et al. [22] proposed a novel algorithm based on the geometrical information of scanning electron microscopic (SEM) images to reconstruct the structure of carbon fiber and simulated the flow properties by using LBM. Caglar et al. [23] developed a computation approach for the permeability of the 3D fibrous porous material based on the circuit analogy and deep learning to speed up numerical computation.

Nonetheless, given that the geometrical characteristic is a major determining factor in transportation, it is necessary to accurately characterize the microstructures of fibrous porous material. However, it is difficult to represent and reconstruct the complex and multiscale structures of fibrous porous media based on Euclidean geometry. Since a great deal of experiments show that the microstructures of the fibrous porous material indicate statistically fractal scaling laws [24,25,26], a few fractal models such as self-similar Sierpinski carpet [27], fractal capillary model [28], Weierstrass–Mandelbrot (W-M) model [29], etc., have been proposed to study the transport properties of the fibrous porous material. Xu et al. [30] developed a pore-scale physical model based on the pore fractal scaling law and predicted the two-phase relative permeability in unsaturated porous media by Monte Carlo simulation. Zhu et al. [31] developed a fractal model for the power-law fluids in fibrous porous material with consideration of electrokinetic effect and calculated the permeability by solving linearized Poisson–Boltzmann and Navier–Stokes equations. Xiao et al. [32] proposed a fractal solution for the Kozeny–Carman (KC) constant and permeability of the fibrous porous material, which is made up by solid particles and porous fibers. Xu et al. [33] developed a simple multi-scale mathematical method by synthesizing the implicit periodic surface model and the W-M fractal model to reconstruct the 3D structure of porous metal fiber sintered felt and discussed the effects of the surface topography and fractal dimension on the gas permeability. Shou et al. [34] proposed a difference-fractal model for the permeability of viscous flow through fibrous porous material by using difference approach and proved that the permeability greatly depends on the maximum pore scale.

Although fractal geometry theory has been proposed to study the fluid flow through the fibrous porous material, the relevant parameters, including fiber orientation, anisotropic, flow tortuosity, etc., still need to be adjusted based on the specific fibrous structures [35,36,37]. Therefore, in order to address these deficiencies, a new pore-scale model based on fractal geometry is explained in this paper to derive the through-plane permeability of anisotropic fibrous porous material, where an equilateral triangle RUC and the capillary bundle model are combined. In addition, the analytical expression of the average tortuosity in fibrous porous material is presented based on two kinds of representative distribution structure (RDS) of fiber columns. A numerical reconstruction method is also proposed to generate the 3D random structures of fibrous porous material by fractal stochastic method (FSM), and the single-phase fluid flow through transversely isotropic random fibrous porous material was simulated by using the FEM.

## 2. Fractal Model

The random size and distribution of pores in fibrous porous material generally led to two possible flow directions: in-plane and through-plane flow [38]. Thereinto, the through-plane flow through fibrous porous material is an essential phenomenon [39], which has attracted considerable attention [40,41,42]. Thus, a pore-scale fractal model was developed in order to obtain the analytical expression of effective through-plane permeability in this part.

### 2.1. Pore-Scale Model

**Figure 1:**
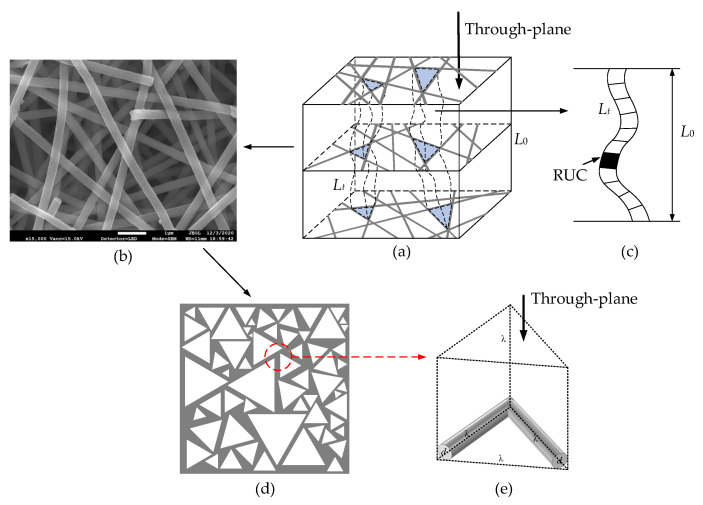
Pore-scale model: (**a**) 3D REV model; (**b**) SEM image; (**c**) flow capillary; (**d**) stochastic triangle pores; (**e**) equilateral triangle RUC model.

The rectangular fiber RUC model for fibrous porous media was proposed by Plessis [43]. It is the smallest unit cell but illustrates the average geometrical properties of a representative elementary volume (REV) [44,45]. A pore-scale model (Figure 1a) for the through-plane flow through an anisotropic fibrous porous material was firstly developed. Different from the rectangular RUC model [46], an equilateral triangle RUC model that is more realistic is proposed to determine the pore size of the fibrous material. It can be seen from the SEM image (Figure 1b) of a fibrous porous material REV that there is a large number of triangular pores with different sizes, and these triangular pores are formed by fiber columns with stochastic length and direction in parallel planes (Figure 1d). The equilateral triangle RUC model (Figure 1e) is assumed in each layer of fibrous porous material. The representative and actual length of the through-plane flow capillary are *L*_0_ and *L_t_*, as shown in Figure 1c, and the symbols *λ* and *d* represent the pore size and the diameter of fiber column, respectively.

### 2.2. Fractal Pore

The pores in fibrous porous material have been proven to show statistically fractal scaling laws [24,25,26], and for simplicity, the pores of model are considered to be ideal spherical pores in fibrous porous material [47]. Thus, the size distribution of the spherical pores in a 3D REV model (Figure 1a) is assumed to follow the fractal probability density function (PDF) [48]:

(1) $$f\left(\lambda \right)=D_{f}\lambda_{\min}^{D_{f}}\lambda^{-(D_{f}+1)}$$

where $\lambda_{\min}$ represents the minimum pore size. The fractal dimension of pore *D_f_* can be determined by [48]

(2) $$D_{f}=d_{E}-\frac{\ln\left(\phi \right)}{\ln\left(\lambda_{\min}/\lambda_{\max}\right)}$$

where ϕ is the bulk porosity, $\lambda_{\max}$ is the maximum pore size, and the Euclidean dimension *d_E_* is 2 and 3, respectively, for the two-dimensional plane and three-dimensional space. The mean pore size of fibrous porous material in the 3D space can be calculated as follows:

(3) $$\lambda_{av}=\int_{\lambda_{\min}}^{\lambda_{\max}}\lambda f\left(\lambda \right)\;d\lambda =\frac{\left(D_{f}+1\right)}{D_{f}}\lambda_{\min}$$

where the range of pore fractal dimension in a 2D plane (*D_f_*) is [1,2], and the fractal criterion ${\left(\lambda_{\min}/\lambda_{\max}\right)}^{D_{f}}=0$ was used because the fractal PDF satisfies normalizing condition. Thus, the total pore volume in fibrous porous material can be calculated as follows:

(4) $$V_{p}=\int_{\lambda_{\min}}^{\lambda_{\max}}\frac{\pi }{6}\lambda^{3}dN=\frac{\pi }{6}\frac{\left(1+D_{f}\right)}{\left(2-D_{f}\right)}\lambda_{\max}^{3}\left(1-\phi \right)$$

In an REV, the bulk porosity can be expressed as $\phi =V_{p}/L_{0}^{3}$. Then, according to Equation (4), the representative length is obtained as

(5) L0=λmax[π6(1+Df)(2−Df)(1−ϕ)ϕ]13

As shown in Figure 1d, the random fiber columns determine the shape of staked fiber layers and can be approximated as equilateral triangles on the cross-sectional area perpendicular to through-plane flow direction. Therefore, the total pore area perpendicular to through-plane flow can be calculated as

(6) $$A_{p}=\int_{\lambda_{\min}}^{\lambda_{\max}}\frac{\sqrt{3}}{4}\lambda^{2}dN=\frac{\sqrt{3}}{4}\frac{D_{f}}{\left(2-D_{f}\right)}\lambda_{\max}^{2}\left(1-\phi \right)$$

where ϕ is the surface porosity. The surface porosity in Equation (6) is taken as equal to the bulk porosity in Equation (2), and the cross-sectional area in an REV is $A=A_{p}/\phi$. The maximum pore volume can $V_{\max-pore}$ be expressed as

(7) $$V_{\max-pore}=V_{t}-V_{s}=V_{s}\frac{\phi }{\left(1-\phi \right)}$$

where $V_{t}=V_{s}/\left(1-\phi \right)$ is the total volume. Since the RUC can represent the averaged geometrical properties of an REV [45], the volume of fibers in the maximum pore can be written as $V_{s}=\left(\pi /4\right)d^{2}\left(2\lambda_{av}-\sqrt{3}d\right)$. Based on fractal geometry theory, the shapes of pores with different size in fibrous porous material are similar. Therefore, the largest pore volume corresponds to the maximum pore size. The maximum pore size on the cross-sectional area perpendicular to through-plane flow direction can be deduced from Equation (7):

(8) λmax=d[π3(2λavd−3)ϕ(1−ϕ)]13

### 2.3. Fractal Tortuosity

**Figure 2:**
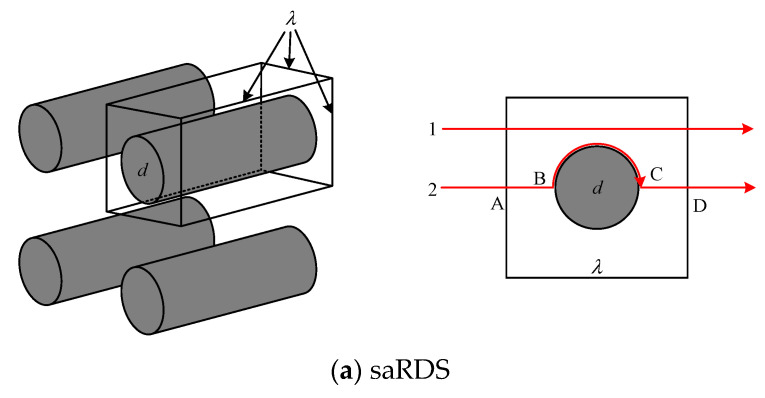

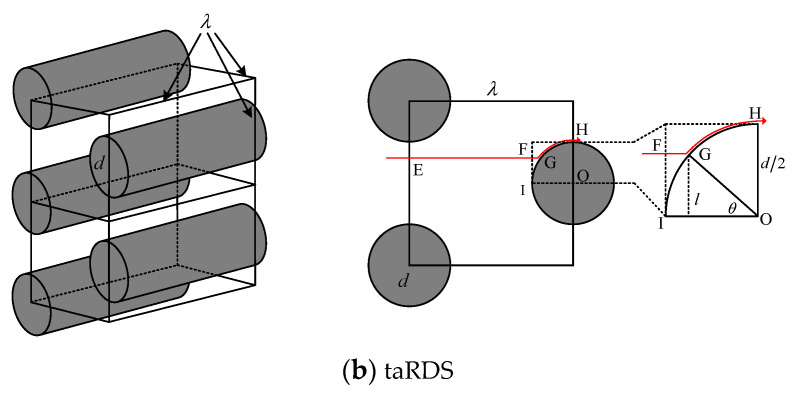
The representative distribution structures of fiber columns: (**a**) square arrangement; (**b**) triangular arrangement.

As shown in Figure 1a,c, the through-plane flow paths can be represented as a bundle of capillaries. The tortuous length of capillaries can be characterized by the fractal scaling law [49]:

(9) $$L_{t}\left(\lambda \right)=\lambda^{\left(1-D_{T}\right)}L_{0}^{D_{T}}$$

where *D_T_* is the tortuosity fractal dimension and can be written as

(10) $$D_{T}=1+\frac{\ln\left(\tau_{av}\right)}{\ln\left(L_{0}/\lambda_{av}\right)}$$

where $\tau_{av}$ is the averaged tortuosity in a porous medium. Since the tortuosity is usually employed to characterize the conducting properties of porous media [50,51], two kinds of RDS of fiber columns were proposed to determine the tortuosity. As shown in Figure 2, the square arrangement (saRDS) and triangular arrangement (taRDS) were used here. The size pore and fiber column are *λ* and *d*, respectively. According to the definition of tortuosity [52] and saRDS in Figure 2a, the tortuosity of streamline 1 is $\tau_{1}\left(\lambda \right)=1$, and the tortuosity of streamline 2 is $\tau_{2}\left(\lambda \right)=\left(l_{AB}+l_{BC}+l_{CD}\right)/\lambda =1+\left(\pi /2-1\right)\left(d/\lambda \right)$. Because the proportion of the straight streamlines decreases, and the proportion of the curved streamlines increases with the increase of fiber column diameter, the averaged tortuosity of saRDS can be obtained by a weighted average of all possible streamlines $\tau_{av}=\sum_{i=1}^{n}a_{i}\tau_{i}$ [53], where $a_{i}$ is the weight value, and $\tau_{i}$ is the tortuosity of the *i^th^* streamline (*i* = 1, 2, 3, …, *n*). Therefore, the average tortuosity of saRDS is calculated as follows:

(11) $$\tau_{I}\left(\lambda \right)=a_{1}\tau_{1}\left(\lambda \right)+a_{2}\tau_{2}\left(\lambda \right)=\tau_{1}\frac{\left(\lambda^{3}-\lambda \pi d^{2}/4\right)}{\lambda^{3}}+\tau_{2}\frac{\lambda \pi d^{2}/4}{\lambda^{3}}=1+\frac{\pi }{4}\left(\frac{\pi }{2}-1\right){\left(\frac{d}{\lambda }\right)}^{3}$$

Based on the weight-average method and taRDS shown in Figure 2b, the averaged tortuosity of taRDS can be expressed as

(12) $$\tau_{II}\left(\lambda \right)=\tau_{EGH}\left(\lambda \right)=\left[\left(\lambda -\frac{d}{2}\right)\tau_{EF}\left(\lambda \right)+\frac{d}{2}\tau_{FGH}\left(\lambda \right)\right]\left(\frac{1}{\lambda }\right)$$

where $\tau_{EF}\left(\lambda \right)=1$. The tortuosity of streamline *FGH* is given by [54]:

(13) $$\tau_{FGH}\left(\theta \right)=\left(l_{FG}+l_{GH}\right)/\left(d/2\right)=\left(1+\pi /2\right)-\left[\cos\left(\theta \right)+\theta \right]$$

where θ=arcsin(2l/d) is the angle ∠*GOI* in Figure 2b. Then, the expression for the tortuosity in dotted box is $\tau_{FGH}\left(l\right)=\left(1+\pi /2\right)-\left[\cos\left(\arcsin\left(2l/d\right)\right)+\arcsin\left(2l/d\right)\right]$, and the result of $\tau_{FGH}\left(\lambda \right)$ is calculated by integrating $\tau_{FGH}\left(l\right)$ over 0<L<d/2.

(14) $$\tau_{FGH}\left(\lambda \right)=\int_{0}^{d/2}\tau_{FGH}\left(l\right)dl/\left(d/2\right)=2-\frac{\pi }{4}$$

Therefore, the tortuosity of taRDS is obtained as

(15) $$\tau_{II}\left(\lambda \right)=1+\left(\frac{1}{2}-\frac{\pi }{8}\right)\frac{d}{\lambda }$$

The pores are randomly distributed in fibrous porous material, and the percentage of the square arrangement was assumed to be the same as that of the triangular arrangement. Then, the average tortuosity of RDS can be deduced from Equations (11) and (15) as follows:

(16) $$\tau \left(\lambda \right)=\frac{\left[\tau_{I}\left(\lambda \right)+\tau_{II}\left(\lambda \right)\right]}{2}=1+\frac{\pi }{8}\left(\frac{\pi }{2}-1\right){\left(\frac{d}{\lambda }\right)}^{3}+\left(\frac{1}{4}-\frac{\pi }{16}\right)\frac{d}{\lambda }$$

Based on the fractal scaling law of pore (Equation (1)), the averaged tortuosity of an REV can be written as follows:

(17) $$\tau_{av}=\int_{\lambda_{\min}}^{\lambda_{\max}}\tau \left(\lambda \right)f\left(\lambda \right)d\lambda =1+\frac{\pi }{8}\left(\frac{\pi }{2}-1\right)\frac{\left(1+D_{f}\right)}{\left(4+D_{f}\right)}{\left(\frac{d}{\lambda_{\min}}\right)}^{3}+\left(\frac{1}{4}-\frac{\pi }{16}\right)\frac{\left(1+D_{f}\right)}{\left(2+D_{f}\right)}\left(\frac{d}{\lambda_{\min}}\right)$$

The averaged pore size can be estimated by the RDS. The pore volume of the RDS is $V_{p-RDS}=\lambda_{av}^{3}-\lambda_{av}\pi d^{2}/4$, while the total volume of the RDS is $V_{t-RDS}=\lambda_{av}^{3}$. The porosity of the RDS is $\phi =V_{p-RDS}/V_{t-RDS}$. Therefore, the ratio of fiber size to averaged pore size is

(18) $$\left(d/\lambda_{av}\right)=\sqrt{4\left(1-\phi \right)/\pi }$$

By combining Equations (3), (17) and (18), the averaged tortuosity can be expressed as

(19) $$\tau_{av}=1+\left(\frac{\pi^{2}}{16}-\frac{\pi }{8}\right)\left[\frac{{\left(1+D_{f}\right)}^{4}}{D_{f}^{3}\left(4+D_{f}\right)}\right]{\left[\sqrt{\frac{4\left(1-\phi \right)}{\pi }}\right]}^{3}+\left(\frac{1}{4}-\frac{\pi }{16}\right)\left[\frac{{\left(D_{f}+1\right)}^{2}}{D_{f}\left(D_{f}+2\right)}\right]\sqrt{\frac{4\left(1-\phi \right)}{\pi }}$$

Then, the fractal dimension for tortuosity is obtained by combining Equations (10) and (19) as

(20) DT=1+ln{1+(π216−π8)[(1+Df)4Df3(4+Df)][4(1−ϕ)π]3+(14−π16)[(Df+1)2Df(Df+2)]4(1−ϕ)π}ln{λmaxλminDf(1+Df)[π6(1+Df)(2−Df)(1−ϕ)ϕ]13}

### 2.4. Through-Plane Permeability

The single-phase fluid-flow rate through a curved capillary in fibrous porous material is assumed to satisfy the modified Hagen–Poiseuille equation:

(21) $$q\left(\lambda \right)=G\frac{\Delta P}{L_{t}\left(\lambda \right)}\frac{\lambda^{4}}{\mu }$$

where $G=\sqrt{3}/36$ is the shape factor of the fluid through equilateral triangle capillaries [55], λ is the pore size, μ is the fluid viscosity, ΔP is the pressure drop along the capillary, and *L_t_* is actual length of curved capillary. Therefore, based on Equations (1) and (9), the total flow rate of through-plane flow through the fibrous porous material can be calculated as follows:

(22) $$Q=-\int_{\lambda_{\min}}^{\lambda_{\max}}q\left(\lambda \right)\;f\left(\lambda \right)N_{t}d\lambda =\frac{\sqrt{3}}{36}\frac{\Delta P}{\mu }\frac{A}{L_{0}}\frac{L_{0}^{\left(1-D_{T}\right)}}{A}\frac{D_{f}}{\left(3+D_{T}-D_{f}\right)}\lambda_{\max}^{\left(3+D_{T}\right)}$$

where $N_{t}={\left(\lambda_{\max}/\lambda_{\min}\right)}^{D_{f}}$ is the total number of pores in a cross-sectional area in an REV. According to Darcy’s law $Q=\frac{K}{\mu }A\frac{\Delta P}{L_{0}}$, the effective through-plane permeability can be expressed as

(23) $$K=\frac{\sqrt{3}}{36}\frac{L_{0}^{\left(1-D_{T}\right)}}{A}\frac{D_{f}}{\left(3+D_{T}-D_{f}\right)}\lambda_{\max}^{\left(3+D_{T}\right)}$$

Inserting Equations (5), (6), (8), (18) and (20) into (23), the dimensionless through-plane permeability of an anisotropic fibrous porous material is obtained as

(24) $$\frac{K}{d^{2}}=\frac{1}{9}\frac{\left(2-D_{f}\right)}{\left(3+D_{T}-D_{f}\right)}\frac{\phi }{\left(1-\phi \right)}{\left[\frac{\pi }{6}\frac{\left(1+D_{f}\right)}{\left(2-D_{f}\right)}\frac{\left(1-\phi \right)}{\phi }\right]}^{\left(1-D_{T}\right)/3}{\left\{\frac{\pi }{\sqrt{3}}\left[\sqrt{\frac{\pi }{\left(1-\phi \right)}}-\sqrt{3}\right]\frac{\phi }{\left(1-\phi \right)}\right\}}^{2/3}$$

It can be found from Equation (24) that the through-plane permeability depends on both fiber column and pore structure; it is a function of fiber column size, porosity, and fractal dimensions for pore and tortuosity. The derivation details of pore size and tortuosity are provided in Appendix A.1 and Appendix A.2, respectively.

## 3. Numerical Simulation

**Figure 3:**
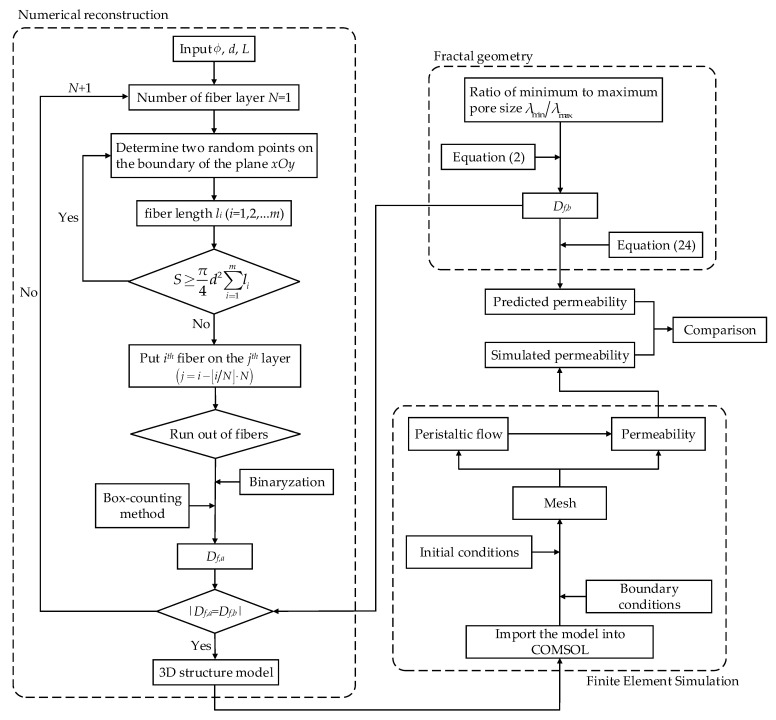
Flowchart of fractal reconstruction and FEM simulation.

A FSM was developed to numerically reconstruct the microstructure of the fibrous porous material, and the single-phase fluid flow through the digital 3D fibrous porous material was studied by FEM. The effective through-plane permeability calculated by the numerical simulation was compared to that by Equation (24) in order to verify the accuracy of the proposed fractal model in Section 2. The procedures for the reconstruction and simulation of 3D random fibrous porous material are illustrated in Figure 3.

The following assumptions were established in the reconstruction by FSM: (1) fiber columns are cylinders with uniform diameter that are also straight and infinite in length; (2) fiber columns in the same layer are allowed intersection while touching but not intersecting between layers; (3) fiber layers are stacked along the *z*-direction, and each fiber layer is parallel to the plane *xOy*.

The procedures of FSM include: (1) input REV size *L*, fiber column diameter *d*, and porosity ϕ and calculate the fractal dimension of cross-sectional pore *D_f,b_* based on Equation (2); (2) start the number of fiber layers from *N* = 1; (3) select two random points on the boundary of the plane *xOy* and generate a cylindrical fiber column, then calculate the fiber column length *l_i_* (*i* = 1, 2, 3, …, *m*) and measure the fiber volume by $\frac{\pi }{4}d^{2}\sum_{i=1}^{m}l_{i}$ until it reaches the value of $S=NdL^{2}\left(1-\phi \right)$; (4) put *i^th^* (*i* = 1, 2, 3, …, *m*) fiber column on the *j^th^* layer (j=i−⌊i/N⌋⋅N); (5) binarize the cross-sectional area obtained in Step 4 and use box-counting method to calculate the fractal dimension of cross-sectional *D_f,a_*; (6) return to Step 2 and set the number of fiber layers to *N* + 1 if $D_{f,a}\neq D_{f,b}$; (7) finish reconstruction process and output the 3D structural model of fibrous porous material. The reconstruction procedures of the concrete example of fibrous porous material are shown in Appendix A.3.

For the incompressible single-phase flow of Newtonian fluid in a 3D fibrous porous material, the governing equations are

**Figure 4:**
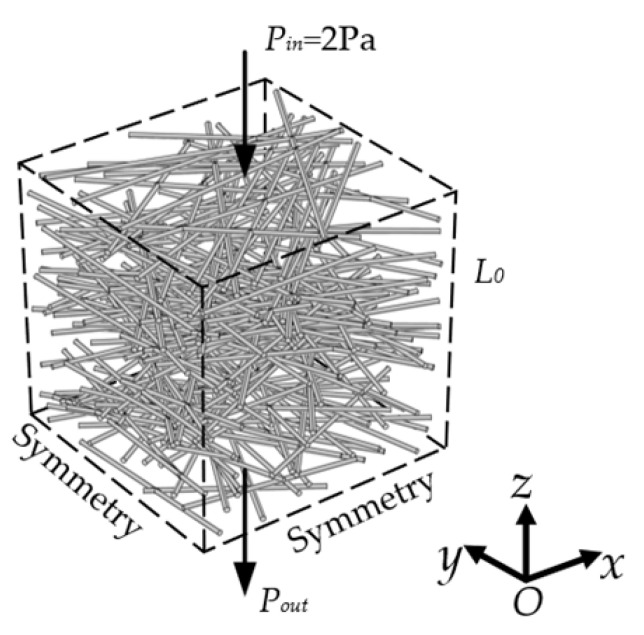
The physical model of 3D fibrous porous material with boundary conditions.

(25) ∇⋅u=0

(26) $$-\nabla p+\nabla \cdot \mu \left(\nabla u+\nabla u^{T}\right)=0$$

where *p*, **u**, and μ represent the pressure, velocity field, and dynamic viscosity of the fluid, respectively. As shown in Figure 4, the pressure drop of 2 Pa is applied between inlet and outlet, and the symmetric boundary condition is used for other faces. The fluid dynamic viscosity and density are 0.001 kg/(m·s) and 1000 kg/m^3^, respectively. The peristaltic flow module in COMSOL Multiphysics was adopted, and the independency of grid density was also examined.

In order to validate the present fractal model, the through-plane permeability by FEM under the same ratio of minimum to maximum of pore size ($\lambda_{\min}/\lambda_{\max}=0.4$) and the dimensionless permeability by fractal model (Equation (24)) were compared and are listed in Table 1. It should be noted that the through-plane permeability error between fractal model and numerical is within 5%, which indicates that the fractal model agrees well with numerical simulation.

**Figure 5:**
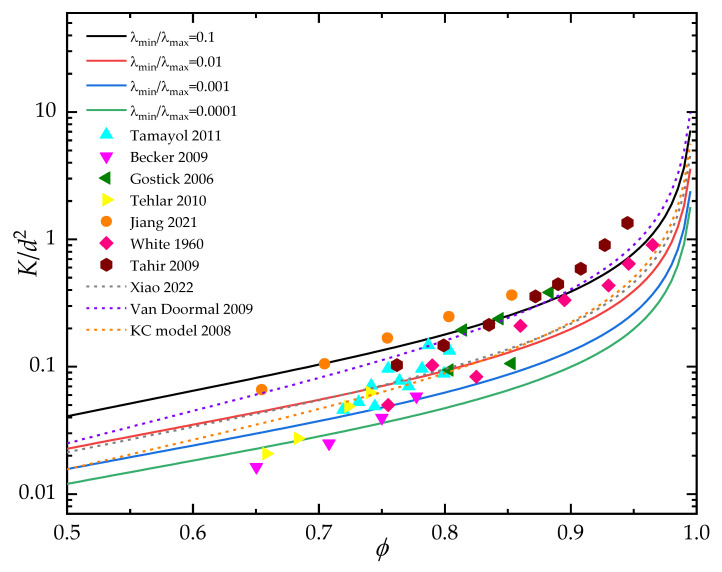
Comparison of predicted through-plane permeability with available experiment data and empirical formulas [56,57,58,59,60,61,62,63,64,65].

The predictions of the through-plane permeability by the present fractal model (Equation (24)) were also compared with available experimental data [56,57,58,59,60,61,62] and common empirical formulas [63,64,65]. As shown in Figure 5, the dimensionless through-plane permeability increases with increased porosity. It can be clearly seen in Figure 5 that the through-plane permeability by the present fractal model is in good agreement with available empirical formulas over the whole range of porosity. For the relative low porosity (0.65<ϕ<0.85), the present fractal model shows better agreement with experimental data compared with that of empirical formulas. However, at the relative high porosity (ϕ>0.85), the predicted through-plane permeability is slightly lower than that of the experimental data. It can be ascribed to the small number of fiber columns at high porosity, which induces the increases of the ratio of minimum to maximum pore size ($\lambda_{\min}/\lambda_{\max}$) of a 3D fibrous porous material and the $\lambda_{\min}/\lambda_{\max}>0.1$ in high porosity. Thus, the flow resistance of the present mathematical model is generally higher than that of the experimental data.

## 4. Results and Discussion

**Figure 6:**
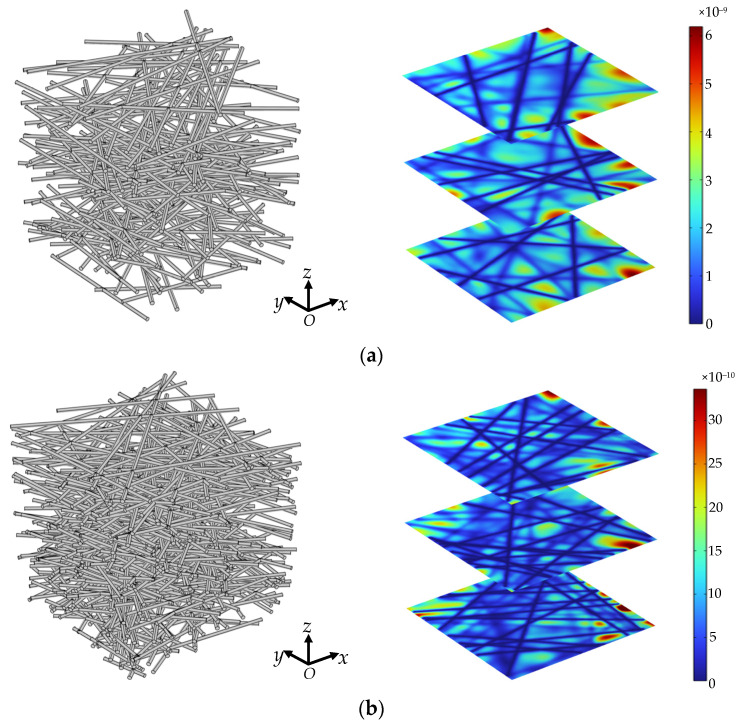
3D structure and velocity distribution (m/s) of random fibrous porous material with different porosity: (**a**) ϕ=0.95; (**b**) ϕ=0.90.

Figure 6 shows the structure of 3D fibrous porous material with porosity of 0.95 and 0.90 by FSM and the velocity distribution on the cross-sectional area perpendicular to through-plane fluid flow by FEM simulation. Driven by the identical pressure drop, the pore spaces become more wide-open at high porosity, and the average velocity on the cross-sectional area is large. As the porosity decreases, the capillary tortuosity increases, and there is a certain blocking effect of fiber columns on fluid flow, resulting in lower and more uniform fluid velocity in the pore space.

**Figure 7:**
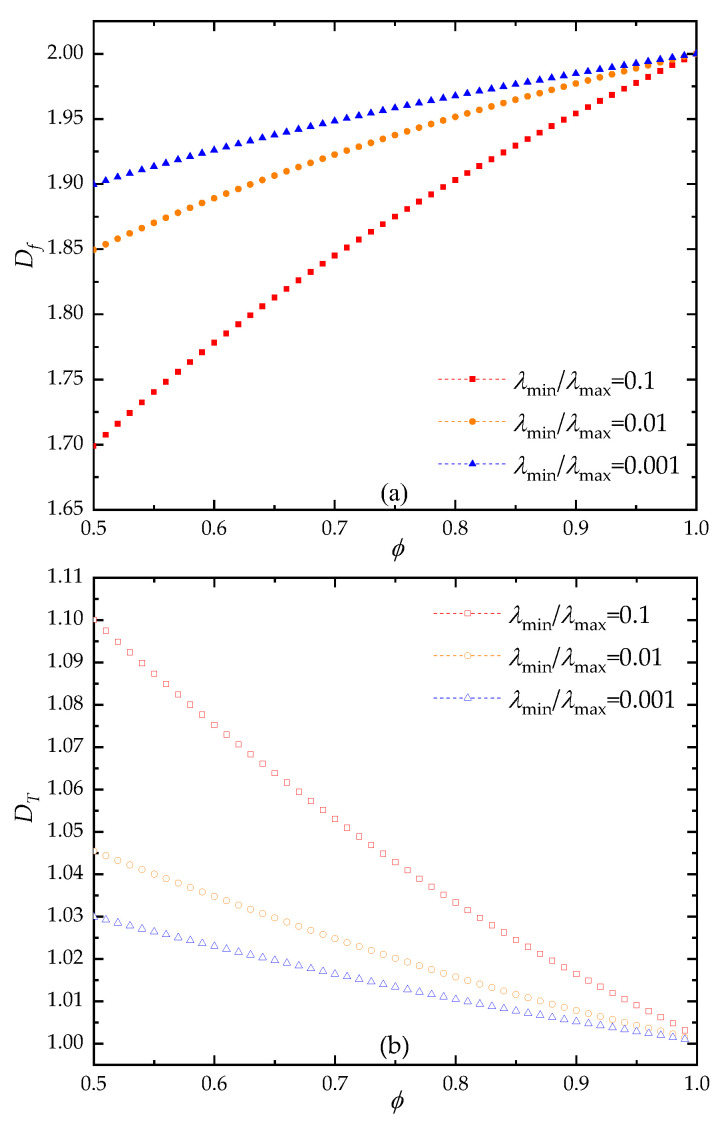
The relationship between fractal dimension and porosity: (**a**) pore fractal dimension; (**b**) tortuosity fractal dimension.

**Figure 8:**
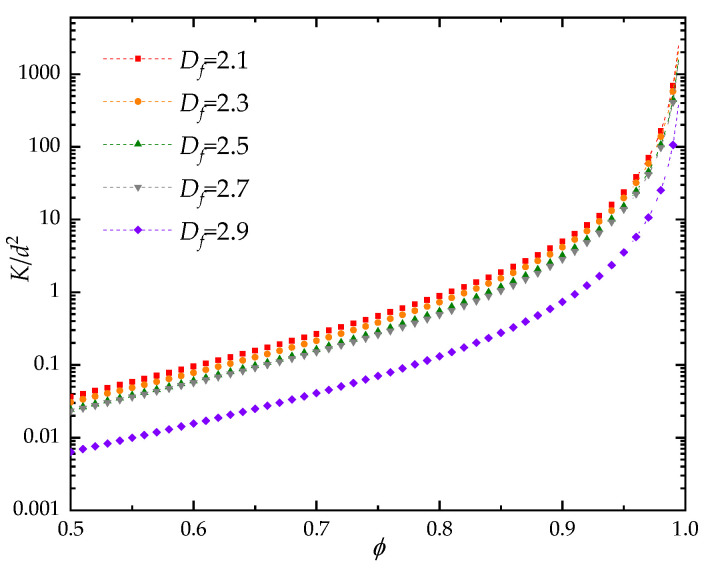
The effect of pore fractal dimension on the dimensionless through-plane permeability of fibrous porous material.

**Figure 9:**
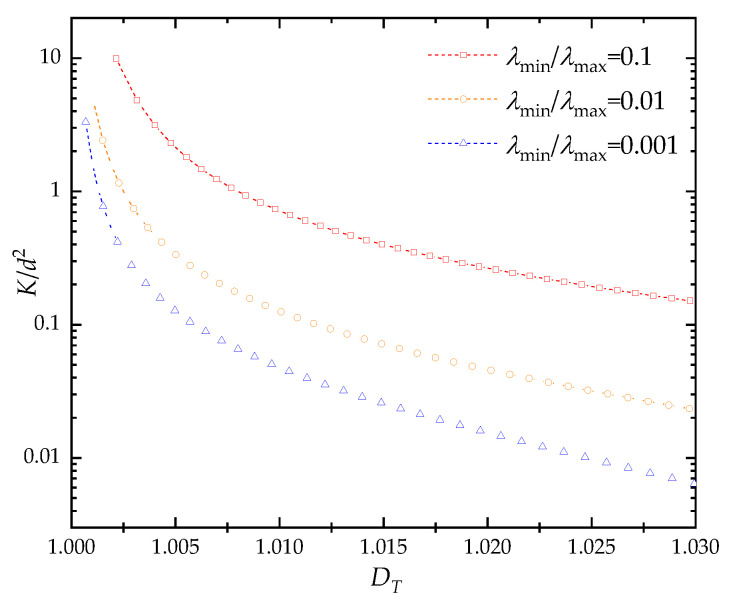
The effect of tortuosity fractal dimension on the dimensionless through-plane permeability of fibrous porous material.

The influences of geometrical parameters on the dimensionless through-plane permeability of fibrous porous material were also examined. As shown in Figure 7, at certain pore size range ($\lambda_{\min}/\lambda_{\max}$), the pore fractal dimension is positively correlated with the porosity, while the tortuosity fractal dimension is negatively correlated with the porosity. With the increase of pore size range (ratio of minimum to maximum pore size decreases), the pore structure becomes more complicated, and the portion of small pores increase. Thus, the pore and tortuosity fractal dimensions increase and decreases with the increase of pore size range under fixed porosity, respectively. It can be found in Figure 8 that the through-plane permeability decreases as the pore fractal dimension increases at certain porosity. This is because the complexity of the pores increases as the fractal dimension for pore distribution under fixed porosity, which induces the increase of flow resistance. As shown in Figure 9, the dimensionless through-plane permeability significantly decreases with the increase of tortuosity fractal dimension under certain pore size range. It can be explained as that the increased porosity reduces the likelihood of fiber columns’ intersection; thus, the tortuosity of fibrous porous material decreases with the increment of pore space. Meanwhile, the fiber columns in a 3D space generally increase the blocking effect on fluid flow with increased tortuosity fractal dimension, and when the pore size range increases (the value of $\lambda_{\min}/\lambda_{\max}$ decreases), more fiber columns with similar orientations are used to reconstruct the fibrous porous material. Therefore, the through-plane permeability decreases as the pore size range increases. It should be noted that the nonlinear phenomena in fluid flow through the fibrous material may have correlation with the anisotropic properties [66].

## 5. Conclusions

In this paper, by combining the equilateral triangle RUC model and fractal capillary bundle model, a new pore-scale model was developed to predict the effective through-plane permeability of the anisotropic fibrous porous materials, and a numerical reconstruction method is also proposed for the reconstructed 3D random structure of fibrous porous material. The analytical expressions for the effective through-plane permeability and average tortuosity were proposed, and the FEM was applied to study the flow field of the 3D anisotropic fibrous porous material generated by FSM. The proposed fractal model was validated by comparing with numerical simulation and available experimental as well as common empirical formulas. The results indicate that: (1) The error of the effective through-plane permeability by fractal model and numerical simulation is within 5%, and the fractal model agrees well with available experimental data and common empirical formulas. (2) The through-plane permeability is positively correlated with the porosity. (3) The through-plane permeability decreases with the increase of fractal dimension of pore distribution and the tortuosity fractal dimension. The proposed fractal model and numerical simulation for fluid flow through anisotropic fibrous material may help in understanding the structural properties and transport mechanisms of fibrous porous material. The present results may provide a useful basis for the applications of fibrous porous material in thermal energy storage, fuel cells, and aerospace, etc. However, it should be pointed out that more complications, such as holistic anisotropy, connectivity of pores, etc., can be included to further improve the accuracy of the reconstruction method and mathematical model.

## Figures and Tables

**Table 1:** Comparison between fractal model and numerical simulation ($\lambda_{\min}/\lambda_{\max}=0.4$ ).

Porosity	Numerical Model	Equation (2)	Surface *D_f_*	Simulated *K*/*d*^2^	Predicted *K*/*d*^2^	Error of *K*/*d*^2^
0.95	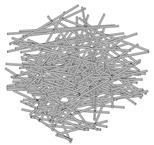	1.9440	1.9462	1.948825	2.01195	3.239%
	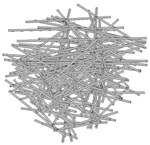		1.9452	1.996975		0.749%
	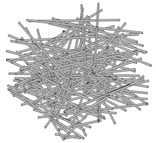		1.9496	1.925575		4.486%
0.90	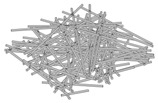	1.8850	1.8922	0.813475	0.8313	2.191%
	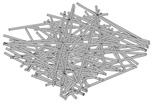		1.8802	0.852175		2.449%
	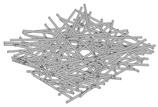		1.8783	0.8563		2.919%
0.85	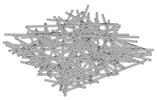	1.8226	1.8284	0.45715		0.702%
	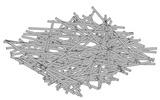		1.8349	0.4474	0.46036	2.897%
	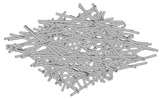		1.8168	0.46995		2.041%

**Figure A1:**
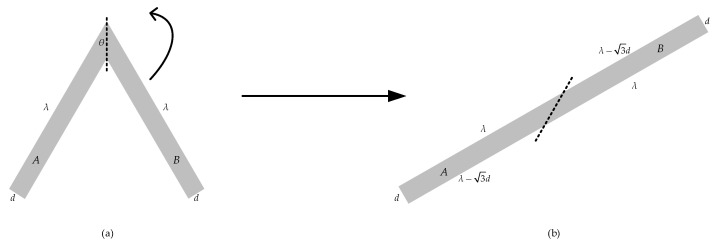
The top view of fiber column in RUC: (**a**) original fiber; (**b**) fiber after segmentation and rotation.

**Figure A2:**
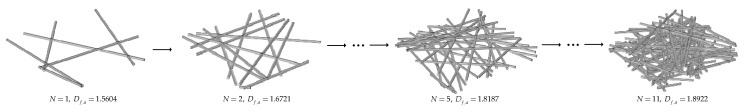
The example of 3D fiber reconstruction.

## Data Availability

All data are contained within the paper, and a report of any other data is not included.

## Appendix A

### Appendix A.1. Pore Size

#### Appendix A.1.1. Average Pore Size

According to Equations (1) and (2), the mean pore size of the fibrous porous material in a 3D space can be calculated as

(A1) $$\lambda_{av}=\int_{\lambda_{\min}}^{\lambda_{\max}}\lambda f\left(\lambda \right)\;d\lambda =\frac{D_{f,3}}{\left(D_{f,3}-1\right)}\lambda_{\max}\left[\frac{\lambda_{\min}}{\lambda_{\max}}-{\left(\frac{\lambda_{\min}}{\lambda_{\max}}\right)}^{D_{f,3}}\right]$$

where the range of pore dimension in a 3D space *D_f,3_* is [2,3]. Because the fractal PDF satisfies normalizing condition, the fractal criterion ${\left(\lambda_{\min}/\lambda_{\max}\right)}^{D_{f,3}}=0$ was inserted into Equation (A1). Then, the mean pore size can be simplified as

(A2) $$\lambda_{av}=\frac{D_{f,3}}{\left(D_{f,3}-1\right)}\lambda_{\min}$$

According to the fractal theory [48], the pore fractal dimensions in the 2D and 3D spaces satisfy $D_{f,3}=D_{f,2}+1$. Thus, the Equation (A2) can be written as follows:

(A3) $$\lambda_{av}=\frac{\left(D_{f,2}+1\right)}{D_{f,2}}\lambda_{\min}$$

where the range of pore dimension in a 2D cross-sectional area *D_f,2_* is [1,2]. In the present work, the 2D pore fractal dimension was used (*D_f_* = *D_f,2_*). Therefore, the mean pore size can be written as $\lambda_{av}=\left[\left(D_{f}+1\right)/D_{f}\right]\lambda_{\min}$.

#### Appendix A.1.2. Maximum Pore Size

As shown in Figure 1d, the random fiber columns determine the shape of staked fiber layers and can be approximated as equilateral triangles in the cross-sectional area perpendicular to through-plane flow direction. Based on fractal geometry theory, the shapes of pores with different size in fibrous porous material are similar. Therefore, the largest pore volume corresponds to the maximum pore size. The maximum pore volume can be expressed as

(A4) $$V_{\max-pore}=\frac{\sqrt{3}}{4}\lambda_{\max}^{2}\cdot \lambda_{\max}=V_{t}-V_{s}=V_{s}\frac{\phi }{\left(1-\phi \right)}$$

where $V_{t}=V_{s}/\left(1-\phi \right)$ is the total volume. As shown in Figure A1, after segmentation and rotation, the volume of fibers $V_{s}$ is given by

(A5)
$$V_{s}=\frac{\pi }{4}d^{2}\left(2\lambda_{av}-\sqrt{3}d\right)$$

The maximum pore size in the cross-sectional area perpendicular to through-plane flow direction can be deduced from Equations (A4) and (A5):

(A6) λmax=d[π3(2λavd−3)ϕ(1−ϕ)]13

### Appendix A.2. Fractal Tortuosity

#### Appendix A.2.1. The Square Arrangement (asRDS)

For the tortuosity of streamline 1 in Figure 2a can be expressed as

(A7) $$\tau_{1}\left(\lambda \right)=1$$

According to the definition of tortuosity, the tortuosity of streamline 2 can be expressed as

(A8) $$\tau_{2}\left(\lambda \right)=\left(l_{AB}+l_{BC}+l_{CD}\right)/\lambda =1+\left(\pi /2-1\right)\left(d/\lambda \right)$$

Because the proportion of the straight streamlines decreases, and the proportion of the curved streamlines increases with the increase of fiber column diameter, the averaged tortuosity of saRDS can be obtained by a weighted average of all possible streamlines [52].

(A9) $$\tau_{av}=\sum_{i=1}^{n}a_{i}\tau_{i}$$

where $a_{i}$ is the weight value, and $\tau_{i}$ is the tortuosity of the *i^th^* streamline (*i* = 1, 2, 3, …, *n*). Therefore, the average tortuosity of saRDS is calculated as

(A10) $$\tau_{I}\left(\lambda \right)=a_{1}\tau_{1}\left(\lambda \right)+a_{2}\tau_{2}\left(\lambda \right)=\tau_{1}\frac{\left(\lambda^{3}-\lambda \pi d^{2}/4\right)}{\lambda^{3}}+\tau_{2}\frac{\lambda \pi d^{2}/4}{\lambda^{3}}=1+\frac{\pi }{4}\left(\frac{\pi }{2}-1\right){\left(\frac{d}{\lambda }\right)}^{3}$$

#### Appendix A.2.2. The Triangular Arrangement (taRDS)

Based on the weight-average method and taRDS shown in Figure 2b, the averaged tortuosity of taRDS can be expressed as follows:

(A11) $$\tau_{II}\left(\lambda \right)=\tau_{EGH}\left(\lambda \right)=\left[\left(\lambda -\frac{d}{2}\right)\tau_{EF}\left(\lambda \right)+\frac{d}{2}\tau_{FGH}\left(\lambda \right)\right]/\lambda$$

where $\tau_{EF}\left(\lambda \right)=1$. The tortuosity of streamline *FGH* is given by [53]

(A12) $$\tau_{FGH}\left(\theta \right)=\left(l_{FG}+l_{GH}\right)/\left(d/2\right)=\left(1+\pi /2\right)-\left[\cos\left(\theta \right)+\theta \right]$$

where θ=arcsin(2l/d) is the angle ∠*GOI* in Figure 2b. Then, the expression for the tortuosity in dotted box can be expressed as

(A13) $$\tau_{FGH}\left(l\right)=\left(1+\pi /2\right)-\left[\cos\left(\arcsin\left(2l/d\right)\right)+\arcsin\left(2l/d\right)\right]$$

Moreover, the result of $\tau_{FGH}\left(\lambda \right)$ is calculated by integrating $\tau_{FGH}\left(l\right)$ over 0<L<d/2.

(A14) $$\tau_{FGH}\left(\lambda \right)=\int_{0}^{d/2}\tau_{FGH}\left(l\right)dl/\left(d/2\right)=2-\frac{\pi }{4}$$

Therefore, the tortuosity of taRDS is obtained as

(A15) $$\tau_{II}\left(\lambda \right)=1+\left(\frac{1}{2}-\frac{\pi }{8}\right)\frac{d}{\lambda }$$

The pores are randomly distributed in fibrous porous material, and the percentage of the square arrangement was assumed the same as that of the triangular arrangement. Then, the average tortuosity of RDS can be deduced from Equations (A10) and (A15) as

(A16) $$\tau \left(\lambda \right)=\frac{\left[\tau_{I}\left(\lambda \right)+\tau_{II}\left(\lambda \right)\right]}{2}=1+\frac{\pi }{8}\left(\frac{\pi }{2}-1\right){\left(\frac{d}{\lambda }\right)}^{3}+\left(\frac{1}{4}-\frac{\pi }{16}\right)\frac{d}{\lambda }$$

Based on the fractal scaling law of pores (Equation (1)), the averaged tortuosity of an REV can be written as follows:

(A17) $$\tau_{av}=\int_{\lambda_{\min}}^{\lambda_{\max}}\tau \left(\lambda \right)f\left(\lambda \right)d\lambda =1+\frac{\pi }{8}\left(\frac{\pi }{2}-1\right)\frac{\left(1+D_{f}\right)}{\left(4+D_{f}\right)}{\left(\frac{d}{\lambda_{\min}}\right)}^{3}+\left(\frac{1}{4}-\frac{\pi }{16}\right)\frac{\left(1+D_{f}\right)}{\left(2+D_{f}\right)}\frac{d}{\lambda_{\min}}$$

By combining Equations (3), (11) and (A17), the averaged tortuosity can be expressed as

(A18) $$\tau_{av}=1+\left(\frac{\pi^{2}}{16}-\frac{\pi }{8}\right)\left[\frac{{\left(1+D_{f}\right)}^{4}}{D_{f}^{3}\left(4+D_{f}\right)}\right]{\left[\sqrt{\frac{4\left(1-\phi \right)}{\pi }}\right]}^{3}+\left(\frac{1}{4}-\frac{\pi }{16}\right)\left[\frac{{\left(D_{f}+1\right)}^{2}}{D_{f}\left(D_{f}+2\right)}\right]\sqrt{\frac{4\left(1-\phi \right)}{\pi }}$$

### Appendix A.3. Numerical Reconstruction

The reconstruction procedures of the concrete example of fibrous porous material are shown in Figure A2. It includes the following steps: (1) calculate the fractal dimension *D_f,b_* = 1.8850 according to Equation (2) with ϕ=0.9 and $\lambda_{\min}/\lambda_{\max}=0.4$; (2) start the number of fiber layers from *N* = 1; (3) generate the 3D structure model of fibrous porous material and calculate the fractal dimension of cross-sectional *D_f,a_*; (4) return to Step 2 and set the number of fiber layers to *N* + 1 if $D_{f,a}\neq D_{f,b}$; (5) finish the reconstruction process until $D_{f,a}=D_{f,b}$.
